# Supplementary material for: Antiretroviral Treatment-Induced Decrease in Immune Activation Contributes to Reduced Susceptibility to Tuberculosis in HIV-1/Mtb Co-infected Persons
Source: Front Immunol. 2021 Mar 5;12:645446. doi: 10.3389/fimmu.2021.645446 (PMC7973093; doi:10.3389/fimmu.2021.645446)
Supplement: Supplementary Figure 1 — RNA sequencing analysis results, showing (A) Volcano plot visualizing the differentially expressed genes identified in RNA sequencing analysis; and (B) Hallmark pathway analysis results. [file Data_Sheet_1.docx]

**Supplementary material**

**Antiretroviral treatment-induced decrease in immune activation contributes to reduced susceptibility to tuberculosis in HIV-1/Mtb co-infected persons**

Katalin A. Wilkinson^1,2^, Deborah Schneider-Luftman^1^, Rachel Lai^3^, Christopher Barrington^1^, Nishtha Jhilmeet^2^, David M Lowe^2,4^, Gavin Kelly^1^, Robert J Wilkinson^1,2,3^

^1^ The Francis Crick Institute, London, NW1 1AT, UK

^2^ Wellcome Centre for Infectious Diseases Research in Africa, Institute of Infectious Diseases and Molecular Medicine, University of Cape Town, Observatory 7925, South Africa;

^3^ Department of Infectious Disease, Imperial College London, W12 0NN, UK

^4^ Institute of Immunity and Transplantation, University College London, NW3 2QG, UK

**Supplementary Figures**

Supplementary Figure 1.

RNA sequencing analysis results, showing (A) Volcano plot visualising the differentially expressed genes identified in RNA sequencing analysis; and (B) Hallmark pathway analysis results.

Supplementary Figure 2.

Change in QFT plasma analytes compared to day 0, at 1M, 3M and 6M of ART. Minus log-transformed p-values for each analyte, resulting from the Limma analysis framework, after BH FDR correction at 1 Month, 3 Months and 6 Months of ART, compared to day 0. Sign represents direction of effect size (negative: decrease from D0 level, positive: increase from D0 level). Red lines: α significance thresholds.

Supplementary Figure 3.

Change in CD4 T cells expressing the chemokine receptors CXCR3, CCR4 and CCR6, determined by flow cytometry analysis in peripheral blood mononuclear cells in a subset of 25 patients from the same cohort, at day 0 and 6 months of ART.

**Supplementary Figure 1A**

Volcano plot visualising the differentially expressed genes identified in RNA sequencing analysis


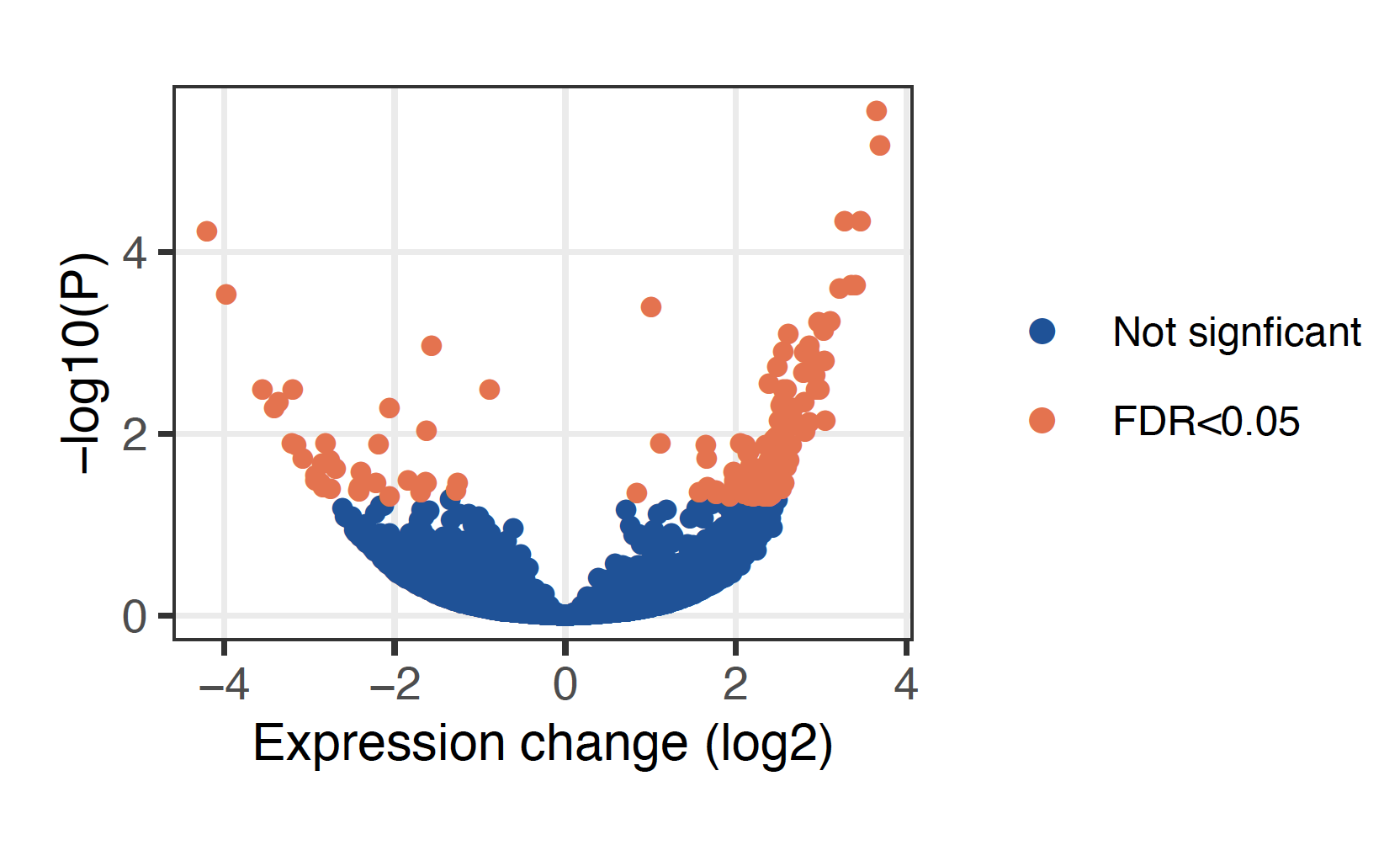


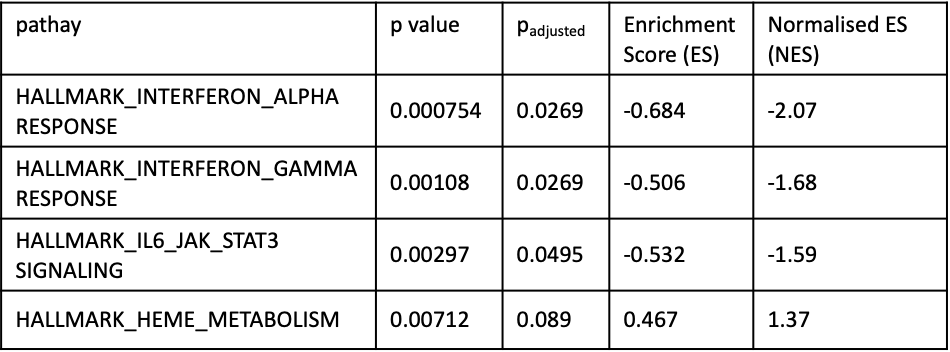

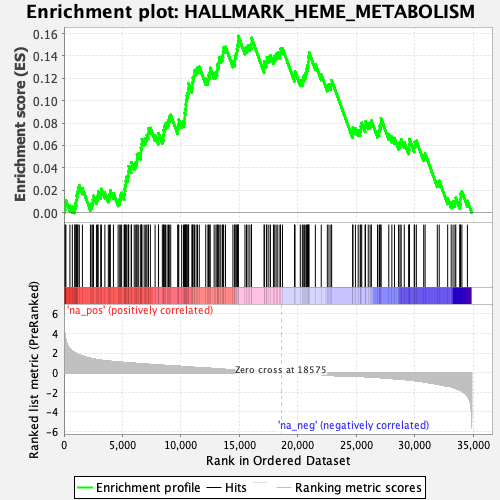

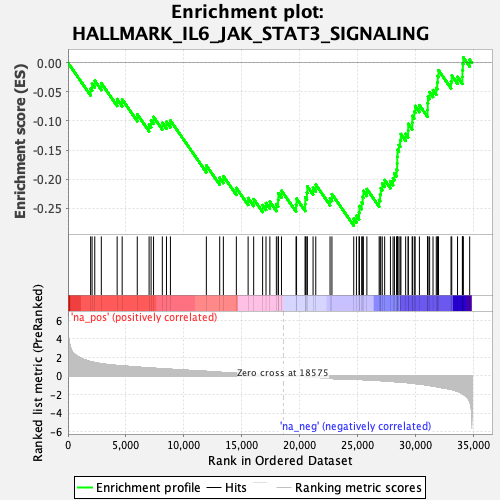

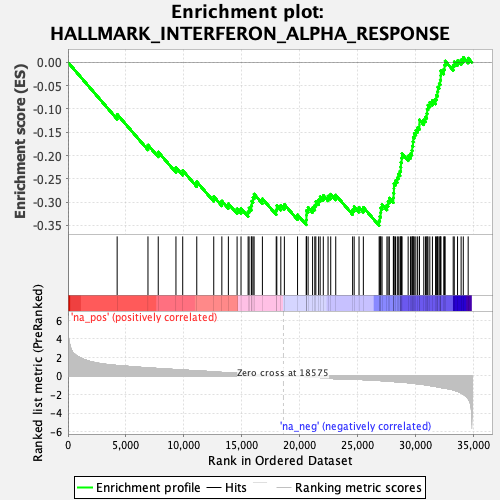

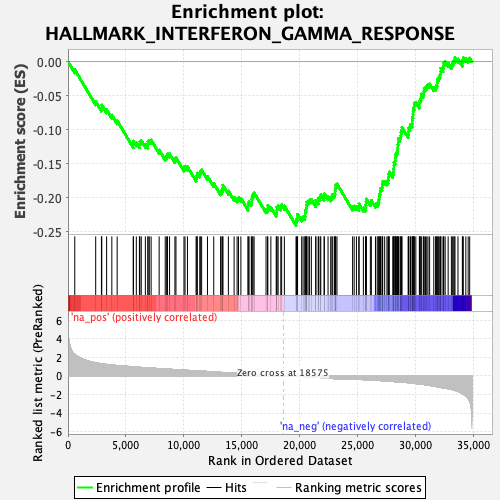
**Supplementary Figure 1B.**  Hallmark pathway analysis results at 6M compared to D0.

**Supplementary Figure 2.** Change in analytes compared to day 0, at 1M, 3M and 6M of ART


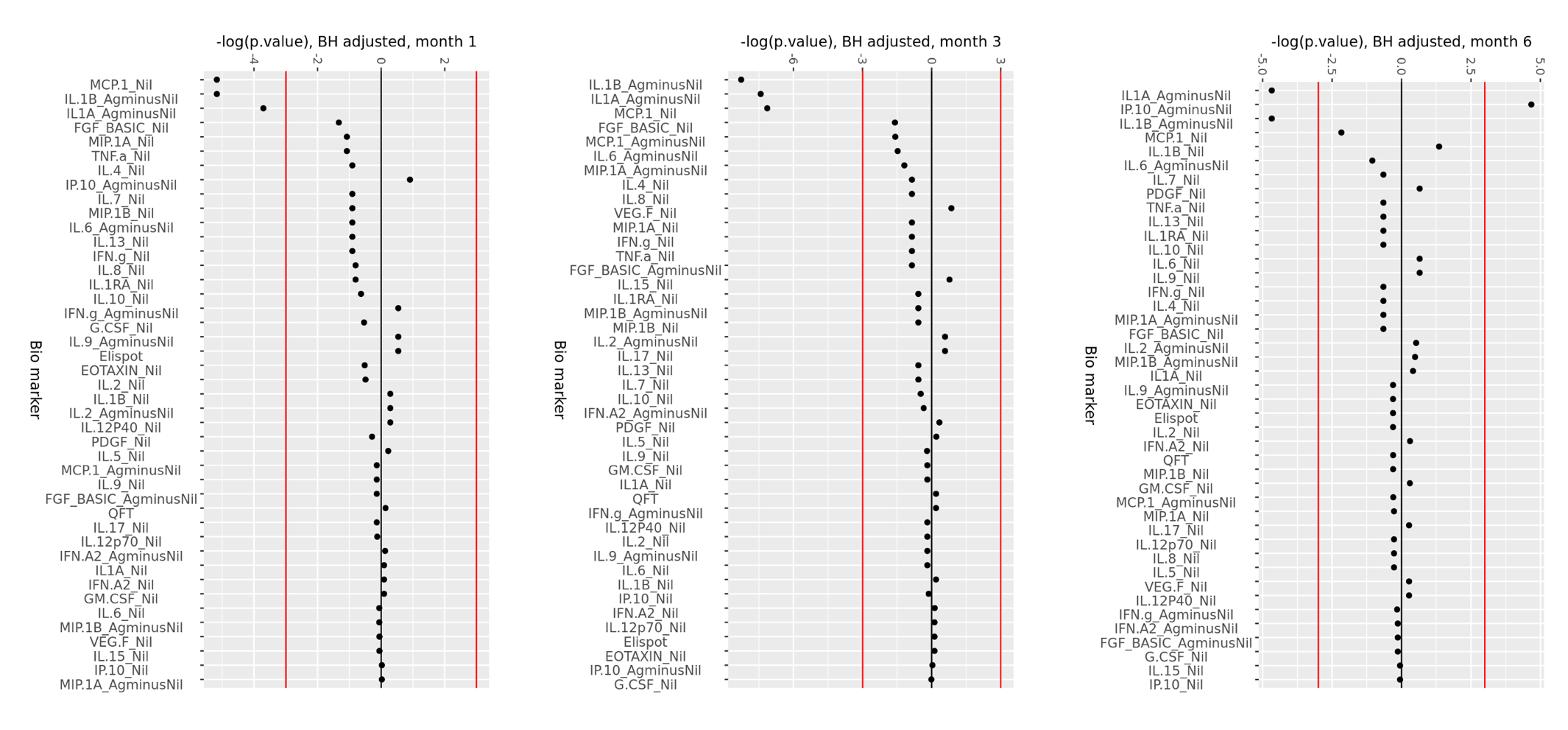


Minus log-transformed p-values for each analyte, resulting from the Limma analysis framework, after BH FDR correction at 1 Month, 3 Months and 6 Months of ART, compared to day 0. Sign represents direction of effect size (negative: decrease from D0 level, positive: increase from D0 level). Red lines: α significance thresholds.

**Supplementary Figure 3.**

Change in CD4 T cells expressing the chemokine receptors CXCR3, CCR4 and CCR6, determined by flow cytometry analysis in peripheral blood mononuclear cells in a subset of 25 patients from the same cohort, at day 0 and 6 months of ART.


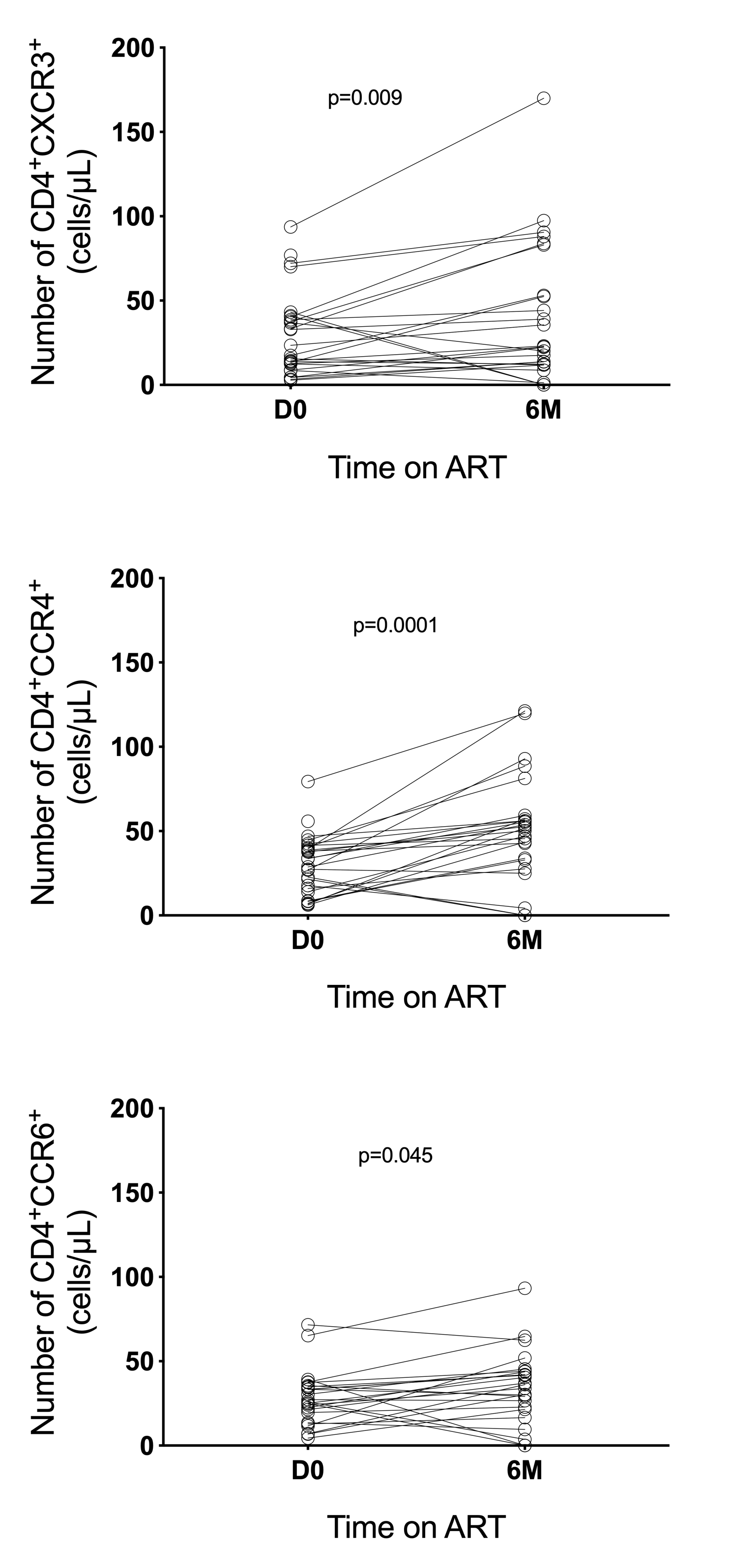


**Supplementary Tables**

**Supplementary Table 1.** Analytes evaluated in the study

| assay | analytes | Sample type (n) |
| --- | --- | --- |
| Human Immune Monitoring 65-plex ProcartaPlex immunoassay (Invitrogen, Thermo Fischer) | APRIL; BAFF; BLC; CD30; CD40L; ENA-78; Eotaxin; Eotaxin-2; Eotaxin-3; FGF-2; Fractalkine; G-CSF; GM-CSF; Gro-alpha; HGF; IFN-alpha; IFN-gamma; IL-10; IL-12p70; IL-13; IL-15; IL-16; IL-17A; IL-18; IL-1alpha; IL-1beta; IL-2; IL-20; IL-21; IL-22; IL-23; IL-27; IL-2R; IL-3; IL-31; IL-4; IL-5; IL-6; IL-7; IL-8; IL-9; IP-10; I-TAC; LIF; MCP-1; MCP-2; MCP-3; M-CSF; MDC; MIF; MIG; MIP-1alpha; MIP-1beta; MIP-3alpha; MMP-1; NGF-beta; SCF; SDF-1alpha; TNF-beta; TNF-alpha; TNF-R2; TRAIL; TSLP; TWEAK; VEGF-A | Plasma (n=38) |
| Human Proinflammatory; cytokine; chemokine; angiogenesis Panel 1, Meso Scale Discovery Inc (Rockville, MD) | IFN-gamma, IL-1beta, IL-2, IL-4, IL-6, IL-8, IL-10, IL-12p70, IL-13, TNF-alpha; IL-1alpha, IL-17A; MIP-1beta, IP-10, MIP-1alpha, MCP-1; VEGF-A, VEGF-C, VEGF-D | Plasma (n=38) |
| Bio-Plex Pro Human Cytokine 27-Plex Immunoassay (BioRad) | IL-1beta, IL-1RA, IL-2, IL-4, IL-5, IL-6, IL-7, IL-8, IL-9, IL-10, IL-12p70, IL-13, IL-15, IL-17, EOTAXIN, FGF BASIC, G-CSF, GM-CSF, IFN-gamma, IP-10, MCP-1, MIP-1alpha, PDGF, MIP-1beta, RANTES, TNF-alpha and VEGF | QFT plasma (n=30)  (Nil and Ag) |
| customized MilliplexTM kits | IL-1alpha, IFN-alpha2 and IL-12p40 | QFT plasma (n=30)  (Nil and Ag) |

**Supplementary Table 2.** Differentially expressed genes revealed by RNA sequencing.

This list is provided in an Excel spreadsheet. The RNAseq data was deposited to Gene Expression Omnibus (GEO) under accession number GSE158208:

<https://www.ncbi.nlm.nih.gov/geo/query/acc.cgi?acc=GSE158208>

**Supplementary Table 3.** MSD analysis results

| Analyte ^1^ | day 0 (median) | day 0 (IQR) | 6M (median) | 6M (IQR) | p value ^2^ |
| --- | --- | --- | --- | --- | --- |
| IFN-gamma | 13.45 | 6.9-26.9 | 6.05 | 2.9-12.7 | <0.0001 |
| IP-10 | 1234 | 753-1798 | 375 | 274-543 | <0.0001 |
| IL-17A | 1.28 | 0.72-2.66 | 0.86 | 0.6-1.5 | 0.0103 |
| TNF-alpha | 2.9 | 2.0-4.3 | 1.17 | 0.9-1.5 | <0.0001 |
| MIP-1alpha | 21.3 | 15-47.7 | 11.5 | 9.8-13.7 | <0.0001 |
| MIP-1beta | 64.1 | 45.1-116.4 | 50.8 | 42.5-85.5 | 0.0047 |
| MCP-1 | 132 | 91.6-155.1 | 111.5 | 89.7-131.6 | 0.0289 |
| VEGF-A | 70.9 | 45.5-129.5 | 49 | 22.4-69.9 | <0.0001 |
| VEGF-C | 49.3 | 31.5-69.1 | 39.2 | 27.8-51.8 | 0.0149 |
| VEGF-D | 851 | 672-1150 | 1046 | 811-1418 | 0.0002 |
| IL-2 | 0.22 | 0.1-0.7 | 0.13 | 0.02-0.46 | 0.12 |
| IL-4 |  |  |  |  |  |
| IL-6 | 1.01 | 0.62-1.27 | 0.64 | 0.49-1.16 | 0.0388 |
| IL-8 | 10.5 | 5.3-18.3 | 4.1 | 3.6-6.9 | <0.0001 |
| IL-10 | 0.33 | 0.25-0.48 | 0.13 | 0.1-0.27 | 0.0002 |
| IL-12p70 | 0.12 | 0.07-0.22 | 0.09 | 0.03-0.13 | 0.0347 |
| IL-13 | 0.82 | 0.55-1.37 | 0.58 | 0.41-1.48 | 0.3965 |

^1^ analyte concentrations expressed as median (IQR) pg/ml; ^2^Wilcoxon matched pairs (n=39).

**Supplementary Table 4.** HIV-1 viral load (VL) and CD4 T-cell counts (CD4) at each time point of the study in n=30 patients included in the QFT plasma analysis.

|  | D0 (Baseline) | Month 1 | Month 3 | Month 6 | P-value |
| --- | --- | --- | --- | --- | --- |
| VL (mean (SD)) | 191,668.53 (315,605.35) | 506.47 (630.34) | 74.10 (66.02) | 507.41 (1,811.05) | <0.001 |
| CD4 (mean (SD)) | 203.83 (117.99) | 273.53 (149.09) | 285.67 (118.76) | 337.60 (137.63) | 0.002 |

**Supplementary Table 5.**

Mean (standard deviation, SD) in pg/ml for all analytes of interest in the study, across each time point in the n=30 patients included in the final analysis. P-value: p-value from group means test for each analyte, where H0 = no change in means across time point.

| mean (SD) | D0, Baseline | Month 1 | Month 3 | Month 6 | p |
| --- | --- | --- | --- | --- | --- |
| IL.1B  Ag-Nil | 4708.11 (6565.77) | 1467.83 (2113.44) | 621.29 (1763.91) | 1685.36 (3121.89) | 0.001 |
| MCP.1 | 2962.56 (1724.55) | 1810.16 (1083.69) | 1699.21 (1225.52) | 2187.01 (1457.96) | 0.003 |
| IL1A  Ag-Nil | 3216.44 (4919.43) | 1163.43 (3171.31) | 425.44 (1206.07) | 963.84 (2747.64) | 0.008 |
| IP.10  Ag-Nil | 26964.24 (36478.84) | 49185.80 (68260.54) | 27893.62 (55218.61) | 75260.40 (91606.46) | 0.017 |
| FGF_BASIC | 365.61 (406.03) | 240.70 (183.56) | 235.04 (168.26) | 300.54 (188.29) | 0.171 |
| MIP.1A  Ag-Nil | 19968.89 (52268.55) | 20230.01 (50426.98) | 1277.49 (16318.18) | 8262.42 (26012.96) | 0.18 |
| IL.4 | 39.76 (89.56) | 18.08 (7.35) | 18.38 (7.97) | 23.79 (16.00) | 0.226 |
| IL.6  Ag-Nil | 48031.93 (72568.76) | 27614.69 (60551.07) | 19766.33 (47979.96) | 22351.93 (57208.77) | 0.261 |
| MCP.1  Ag-Nil | 1816.20 (2326.11) | 1583.14 (1603.22) | 837.45 (1662.22) | 1545.95 (2419.46) | 0.279 |
| MIP.1B  Ag-Nil | 19170.14 (28942.85) | 17149.29 (30706.70) | 6212.26 (19369.55) | 30601.39 (84360.02) | 0.28 |
| PDGF | 4544.61 (2535.39) | 4162.48 (2080.33) | 4959.61 (2499.21) | 5347.80 (2694.88) | 0.284 |
| TNF.a | 1972.18 (4978.72) | 634.87 (756.01) | 957.12 (2369.53) | 940.53 (883.19) | 0.287 |
| IL.1B | 1652.57 (1304.58) | 2041.28 (2808.07) | 1844.11 (1473.68) | 2759.06 (3368.94) | 0.315 |
| MIP.1A | 18353.21 (26909.41) | 10737.14 (13328.16) | 12570.61 (11145.99) | 16454.87 (13891.37) | 0.316 |
| IL.9  Ag-Nil | 56.94 (61.99) | 126.22 (505.43) | 31.28 (77.61) | 6.10 (111.80) | 0.337 |
| VEG.F | 279.88 (289.50) | 242.15 (201.04) | 679.31 (2087.68) | 402.65 (393.96) | 0.393 |
| IL.8 | 44643.46 (90273.23) | 25424.05 (25112.53) | 22987.35 (22977.22) | 38279.87 (59790.61) | 0.398 |
| IL.9 | 166.57 (91.27) | 156.61 (61.51) | 153.80 (67.82) | 189.79 (128.10) | 0.417 |
| IL.7 | 593.92 (1079.99) | 342.39 (422.67) | 426.13 (661.85) | 326.86 (326.59) | 0.424 |
| IL.15 | 97.01 (97.54) | 41.03 (28.40) | 744.61 (3863.76) | 67.62 (93.41) | 0.438 |
| IFN.g | 1299.52 (5347.46) | 305.91 (193.99) | 318.78 (203.09) | 486.22 (748.84) | 0.441 |
| IL.13 | 583.29 (2740.73) | 52.51 (46.72) | 208.99 (816.92) | 71.47 (75.75) | 0.455 |
| IL.1RA | 3151.07 (11137.61) | 1124.37 (3012.41) | 1449.66 (5094.96) | 681.68 (668.11) | 0.463 |
| IL.6 | 11407.80 (13283.86) | 10741.10 (22295.13) | 9970.29 (9960.43) | 16490.83 (22248.46) | 0.492 |
| IL.10 | 137.47 (376.50) | 63.72 (254.96) | 82.12 (351.75) | 27.54 (32.76) | 0.528 |
| FGF_BASIC  Ag-Nil | 116.15 (282.50) | 94.92 (160.83) | 49.75 (133.24) | 108.51 (182.02) | 0.57 |
| IL.17 | 195.90 (208.64) | 145.48 (89.16) | 319.09 (900.80) | 257.17 (381.89) | 0.572 |
| MIP.1B | 21145.36 (36620.25) | 13402.28 (15781.74) | 15713.77 (13875.87) | 16434.82 (15217.00) | 0.6 |
| EOTAXIN | 481.67 (1499.51) | 203.24 (189.62) | 533.38 (1587.78) | 269.98 (266.02) | 0.601 |
| IFN.g  Ag-Nil | 404.24 (2460.48) | 941.18 (2934.91) | 631.37 (2146.20) | 270.51 (965.78) | 0.681 |
| IL1A | 789.52 (1285.82) | 885.11 (2246.29) | 631.82 (920.36) | 1101.11 (1496.70) | 0.708 |
| IFN.A2  Ag-Nil | 218.57 (491.33) | 279.27 (1055.65) | 85.10 (492.44) | 176.34 (364.26) | 0.712 |
| G.CSF | 480.56 (1079.57) | 271.79 (189.92) | 477.51 (1093.35) | 445.77 (501.70) | 0.722 |
| IL.5 | 117.15 (520.83) | 266.03 (1400.08) | 266.12 (1400.11) | 11.26 (24.71) | 0.731 |
| IL.2 | 66.61 (168.74) | 37.33 (111.07) | 54.13 (182.38) | 31.11 (42.01) | 0.75 |
| GM.CSF | 89.16 (349.00) | 107.71 (366.76) | 50.15 (111.41) | 136.75 (347.02) | 0.753 |
| IL.12p40 | 44.87 (103.90) | 69.36 (236.44) | 30.33 (78.32) | 59.95 (124.45) | 0.755 |
| IL.2  Ag-Nil | 95.31 (252.84) | 169.47 (498.24) | 201.21 (635.82) | 209.08 (452.47) | 0.791 |
| IL.12p70 | 182.06 (419.18) | 147.42 (493.06) | 200.31 (709.91) | 82.50 (69.48) | 0.798 |
| IFN.A2 | 444.00 (1015.43) | 492.19 (1040.58) | 493.44 (1032.30) | 580.53 (1138.77) | 0.968 |
| IP.10 | 14704.19 (9004.85) | 14822.17 (21020.31) | 13655.81 (17794.55) | 14526.47 (15720.11) | 0.993 |
